# Change in Atrial Activation Patterns During Narrow Complex Tachycardia: What Is the Mechanism?

**DOI:** 10.19102/icrm.2022.130901

**Published:** 2022-09-15

**Authors:** Şiho Hidayet, Ahmet Korkmaz, Turhan Turan, Abdullah Tunçez, Meryem Kara, Elif Hande Ozcan Cetin, Ozcan Ozeke, Serkan Cay, Firat Ozcan, Dursun Aras, Serkan Topaloglu

**Affiliations:** ^1^Department of Cardiology, Inonu University, Malatya, Turkey; ^2^Department of Cardiology, Health Sciences University, Ankara City Hospital, Ankara, Turkey; ^3^Health Sciences University, Ahi Evren Thoracic and Cardiovascular Surgery Training and Research Hospital, Trabzon, Turkey; ^4^Department of Cardiology, Selcuk University, Konya, Turkey; ^5^Department of Cardiology, Istanbul Medipol University, Istanbul, Turkey

**Keywords:** AVRT, intra-atrial conduction block, mitral isthmus line

## Abstract

A change in the coronary sinus (CS) activation pattern from an eccentric to a concentric pattern during the ablation of an orthodromic reciprocating tachycardia might falsely suggest the presence of a second (septal) accessory pathway (AP) during tachycardia or the successful ablation of the left lateral AP under ventricular pacing despite persistent and unaffected AP conduction. Complete or partial intra-atrial block should be suspected when an abrupt change in the atrial activation sequence is noted during catheter ablation at the posterolateral and lateral aspects of the mitral annulus. The correct anatomical position of the CS catheter plays a vital role in the differential diagnosis of this situation.

## Case presentation

**Figure 1:**
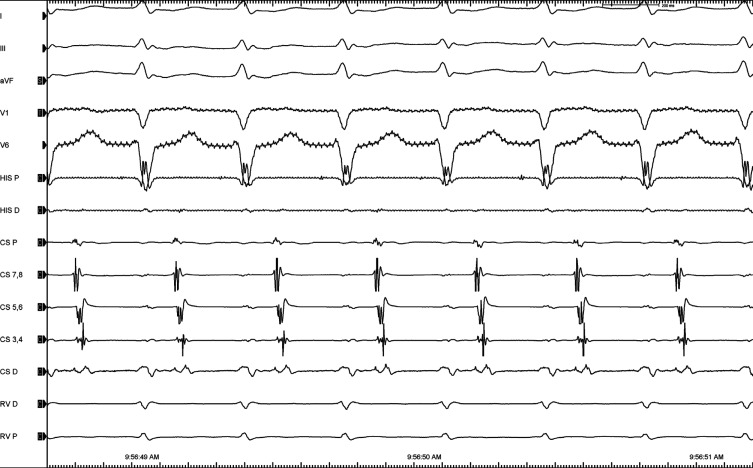
A narrow QRS tachycardia with an apparently concentric retrograde atrial activation.

**Figure 2:**
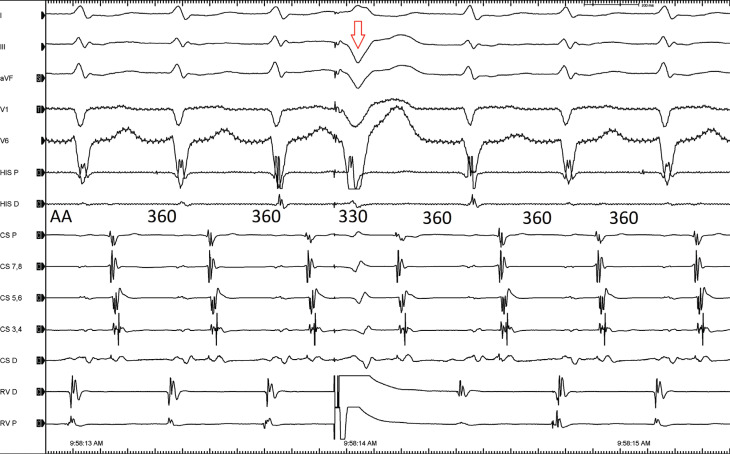
A premature ventricular beat (arrow) timed when the His is refractory readily pre-excites the next atrial electrogram.

**Figure 3:**
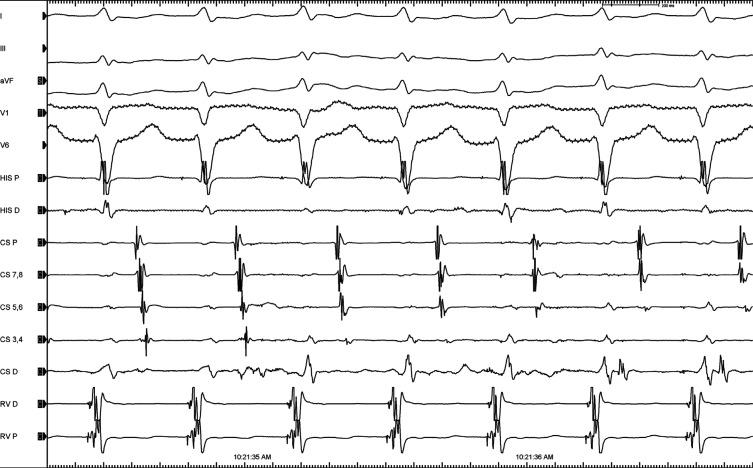
A change in the atrial activation pattern during the narrow complex tachycardia is seen without a change in the tachycardia cycle length.

A 33-year-old male patient with a history of attempted ablation for Wolff–Parkinson–White syndrome utilizing a left lateral pathway was brought to the electrophysiology laboratory because of his recurrent palpitations. A 12-lead electrocardiogram showed no baseline pre-excitation. On electrophysiological study, an anterograde investigation demonstrated a dual atrioventricular nodal physiology. Programmed atrial stimulation easily and reproducibly induced narrow QRS tachycardia **(Figure 1)**. A premature ventricular beat timed when the His was refractory readily pre-excited the next atrial electrogram, confirming the presence of an accessory pathway (AP) **(Figure 2)**. A change in the atrial activation pattern during narrow complex tachycardia was observed without a change in the tachycardia cycle length **(Figure 3)**. What are the mechanisms of tachycardia and change in retrograde atrial activation patterns?

## Discussion

**Figure 4:**
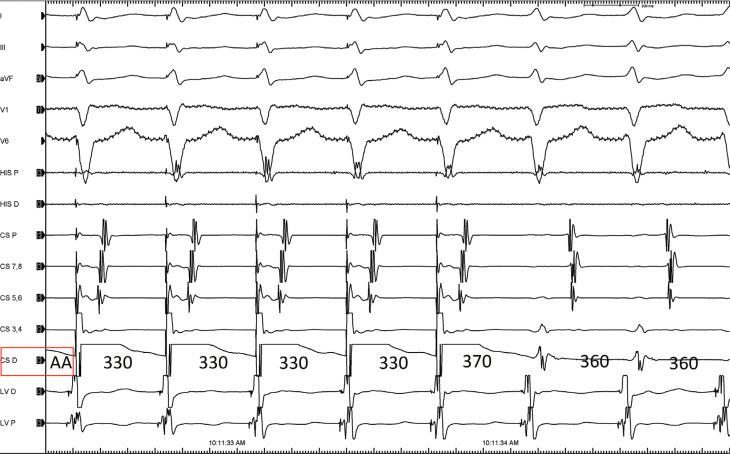
Short post-pacing interval response to atrial entrainment from the distal coronary sinus.

**Figure 5:**
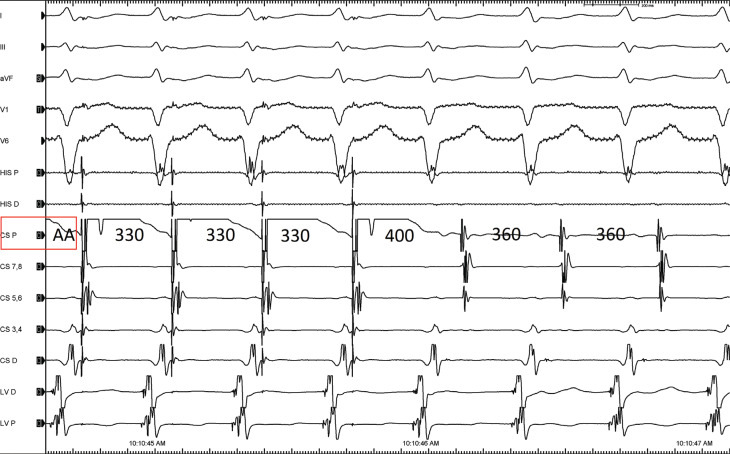
Long post-pacing interval response to atrial entrainment from the proximal coronary sinus.

**Figure 6:**
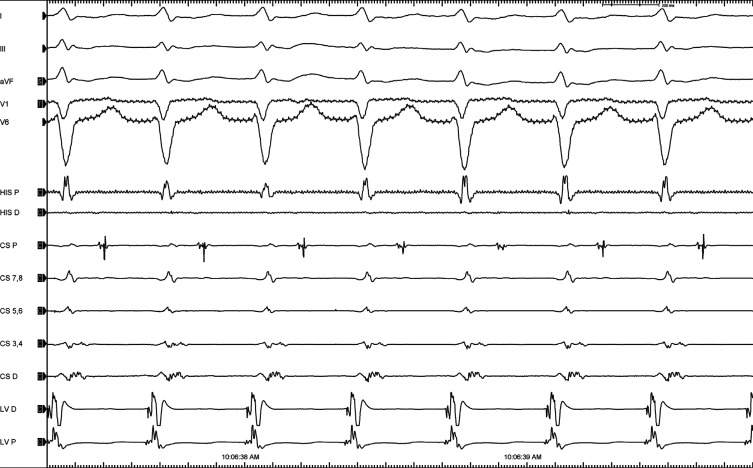
Coronary sinus activation sequence where the coronary sinus catheter is advanced more anterolaterally.

A change in the coronary sinus (CS) activation pattern from an eccentric to a concentric pattern during ablation of the left lateral AP has been previously described.^1–3^ The presence of a second AP or an alternative arrhythmia mechanism should be kept in mind. First, the reversal of eccentric atrial excitation during orthodromic reciprocating tachycardia may falsely suggest the presence of a second (septal) AP. On the other hand, when performing an ablation procedure with ventricular pacing, the operator could be misled to think that successful ablation has been performed due to the change from an eccentric atrial excitation pattern to a concentric pattern. This is despite persistent and unaffected AP conduction. However, if the CS catheter were advanced more anterolaterally **(Figure 5)**, this would identify the persistence of the left lateral AP.^1,2,4–7^ Differential atrial entrainment from both CS distal **(Figure 4)** and proximal **(Figure 5)** sites revealed discordant results. The proximal CS is out of the circuit, but the distal CS is in the re-entry circuit. A possible mechanism of this phenomenon is the occurrence of an intra-atrial conduction block at the mitral isthmus level medial to the insertion of the AP.^3^ This is shown by the distal CS having a sharp, early A with the subsequent reversal from proximal to distal in the remaining electrograms **(Figure 6)**. This is essentially a “signature” for this phenomenon. Luria et al. reported that intra-atrial conduction block may occur in 6.9% of left free-wall AP cases following radiofrequency (RF) delivery along the mitral isthmus, which anatomically is the narrower conductive tissue between the mitral annulus and the left inferior pulmonary vein.^2^ However, intra-atrial conduction block can occur even in a wide mitral isthmus, possibly because of areas of pre-existing functional block or scar.^8^ This phenomenon should be considered when evaluating patients who have undergone previous left lateral AP ablation of another concomitant AP or RF applications in inappropriate areas.^3^
